# The tuberculin skin test in school going adolescents in South India: associations of socio-demographic and clinical characteristics with TST positivity and non-response

**DOI:** 10.1186/s12879-014-0571-7

**Published:** 2014-11-18

**Authors:** Dharma Rao Uppada, Sumithra Selvam, Nelson Jesuraj, Sean Bennett, Suzanne Verver, Harleen MS Grewal, Mario Vaz, TB Trials Study Group

**Affiliations:** St. John’s Emmaus TB Research Initiatives (SETRI), Palamaner, India; St. John’s Research Institute, Bangalore, India; Gilead Sciences, Inc., Foster City, CA USA; KNCV Tuberculosis Foundation, The Hague, The Netherlands; Academic Medical Centre Amsterdam, Amsterdam, The Netherlands; Department of Clinical Science, Infection, Faculty of Medicine and Dentistry, University of Bergen, Bergen, N-5021 Norway; Department of Microbiology, Haukeland University Hospital, Bergen, Norway; Physiology and Head of Health and Humanities, St. John’s Medical College and St. John’s Research Institute, Bangalore, India

**Keywords:** Tuberculosis, Latent tuberculosis infection, Tuberculin skin test, Children

## Abstract

**Background:**

India has generally used 1 TU purified protein derivative (PPD) as opposed to 2 TU PPD globally, limiting comparisons. It is important to assess latent TB infection in adolescents given that they may be a target group for new post-exposure TB vaccines.

The aim of this study is to describe the pattern and associations of tuberculin skin test (TST) responses (0.1 ml 2 TU) in adolescents in South India.

**Methods:**

6643 school-going adolescents (11 to <18 years) underwent TST. Trained tuberculin reader made the reading visit between 48 and 96 hours after the skin test

**Results:**

Of 6608 available TST results, 9% had 0 mm, and 12% ≥10 mm responses. The proportion of TST positive (≥10 mm) was higher among older children, boys, those with a history of TB contact and reported BCG immunization Those with no TST response (0 mm) included younger participants (<14 years), those whose mothers were illiterate and those with a recent history of weight loss. Those of a higher socio-economic status (houses with brick walls, LPG gas as cooking fuel) and those with a visible BCG scar were less likely to be non-responders.

**Conclusion:**

Proportion of non-responders was lower than elsewhere in the world. Proportion of TST positivity was higher in those already exposed to TB and in children who had been BCG immunized, with a zero response more likely in younger adolescents and those with recent weight loss.

**Electronic supplementary material:**

The online version of this article (doi:10.1186/s12879-014-0571-7) contains supplementary material, which is available to authorized users.

## Background

In India, large epidemiological studies have used the tuberculin skin test (TST) to detect tuberculosis (TB) infection, calculate the annual risk of tuberculous infection (ARTI) as a measure of the TB transmission in a community) for different zones of the country, [1] as a means of evaluating the new case detection rate, and for monitoring TB case detection efforts of the Revised National Tuberculosis Control Programme (RNTCP) in the country [1]-[3]. ARTI is computed from the prevalence of infection estimated after tuberculin skin test surveys. In India the TST surveys conducted among the 1–9 years age group in the northern India found that ARTI had declined to 1.1% during 2009 – 10 from 1.9% during 2000 – 01. In the same surveys the prevalence of infection was estimated to have declined from 10.1% to 5.9% [4].

TST data from India has conventionally used a dose of 1TU as opposed to the 2TU dose more universally used [5],[6]. This makes cross-country comparisons with Indian data difficult. While there are no large data on adolescents in India, the proportion of TST positive children in India using a cut off of 20 mm is between 5.9% and 10.5% in 1 to 9 year olds [7]. A TST survey of 5–9 years old children between 2000–2003 in Andhra Pradesh, South India, showed that between 11.6 and 15.5% had no response (0 mm) [2].

Studies in adolescents are important since they may constitute a target population for future vaccines in TB, which aim to prevent active disease in already exposed populations [8],[9] We, therefore conducted this study in school going adolescents between 11 to 18 years, in South India, using a dose of 2 TU of PPD – RT 23 with Tween 80 (Span diagnostics, India), to describe the patterns and socio-demographic and clinical associations of the proportion of TST positive (>10 mm) and non-response (0 mm response). In this study 2 TU was used as the dose. Earlier data by Chadha et al. on BCG un-vaccinated children in India showed no advantage of using 2 TU Vs. 1 TU [6]. However, our decision was based on several considerations. First, the population of this study were mixed with a large proportion of BCG vaccinated adolescents including those with BCG scars (both excluded by Chadha et al.). Second, IUATLD and WHO guidelines now recommend the use of 2 TU [10]. Third, Updated Indian Academy of Pediatrics guidelines, relevant to at least a part of our study population, endorse the use of 2 TU [11]. Finally, the intent was to obtain data that could be compared across countries (most of which use 2TU).

## Methods

### Setting

The present analysis is conducted on data derived from the baseline survey of a prospective cohort of children and adolescents aged between 11 and 18 years attending school or junior college. The aim of the cohort study was to establish the incidence of TB disease during a follow up of enrolled participants over a 2 year period. The cohort study was conducted in the Palamaner area of Chittoor District of Andhra Pradesh in South India from February 2007 to July 2010. The baseline survey was completed between February 2007 and May 2008, the data of which has been used in the present analysis. The annual total case notification rate of TB in the district where the study was conducted was 126 per 100,000 and that for India as a country was 132/100,000 during 2007 - 08 [12].

#### Study design

The present paper is based on a cross-sectional analysis of data obtained at the baseline survey of the prospective, cohort study

### Subjects

6643 adolescents comprising 3441 boys and 3202 girls were recruited into the study, after parental consent and adolescent assent. The total number of eligible adolescents was 12,388; the response rate was 53% for boys and 47% for girls. Participants were excluded if the family had plans to move from the study area in the 2 years following enrollment or were unable to attend the follow-up session for reading of the tuberculin skin test.

### Ethics

Written informed assent from the subjects and consent from the parents/guardians was obtained before the start of enrollment. The study protocol and informed consent forms were reviewed & approved by the Institutional Review Board (IRB) of St John’s Medical College, Bangalore and by an Independent Ethics Committee (IEC) of Aeras, USA. The studies were also approved by the Ministry of Health Screening Committee of the Government of India (No. 5/8/9/52/2006-ECD-I dt. 10.11.2006).

### Assessments

#### Questionnaires

A detailed clinical history of the child including BCG immunization status, history of current or past tuberculosis, history of close contact of more than 8 hours a week with an adult with diagnosed tuberculosis during the last 3 months, and current signs or symptoms of tuberculosis was recorded from the parents/guardians. Adolescents were asked to review the same. Socio-demographic details including the gender and age of the subjects, parental education, whether they were a member of a scheduled caste or scheduled tribe, type of walls of their home and type of cooking fuel used (as surrogates of socio-economic status) and religion were also recorded during the interview.

### Clinical assessments

The presence or absence of a BCG scar was noted. Weight and height were used to compute Body Mass Index (BMI) and derive the BMI-for-age z-scores as a measure of nutritional status using WHO growth reference (WHO, 2007; Growth reference 5–19 years; calculated using WHO – ANTHRO software (version 3.2.2)).

### Tuberculin skin test

Designated, trained, research nurses administered 0.1 ml of tuberculin containing 2 TU Purified Protein Derivative (PPD) of RT 23 with Tween 80 as a stabilizer (SPAN Diagnostic Ltd, Surat, India), on the anterior surface of the forearm about 2 to 4 inches below the elbow. A designated, trained tuberculin reader made the reading visit between 48 and 96 hours after the skin test was administered. The skin test site was inspected in good light. The maximum transverse diameter of the induration was then measured in millimetres, using a transparent ruler calibrated in millimetres. The measurements were recorded and documented along with date and time. While participants who had responses of 0–4 mm TST responses received a second TST within 1–4 weeks of the first and the potential utility of a two-step TST has been reported earlier [13], the present analysis was restricted to the first TST result obtained since a two-step TST is not a standard procedure in India.

### Statistical methods

All data collected were entered into customized data acquisition software using Microsoft SQL for backend operations. Double data entry and a 100% check of the data forms were done to assess missing data and clarity of data prior to data entry. Statistical Package for Social Sciences Version 17.0 (PASW Statistics, 18.0, SPSS Inc, Chicago, IL, USA) was used for the analysis of the data. Chi-square test was used to assess the association of the proportion of TST positive and Non response with socio-demographic and clinical variables. Multivariate analysis was performed using logistic regression to determine the predictors of the proportion of TST positive (TST ≥10 mm) and non-response (TST = 0 mm) adjusted for variables that were significant at the univariate analysis for both the outcomes separately. When evaluating the associations of TST non-response i.e. 0 mm, data was restricted to those who had a TST non response and TST response of 5 mm or greater. This was done to allow for a better separation of groups. However, the results did not qualitatively change when those with a non responses were compared with all those who had a recordable TST response (TST >0 mm). Since a large number of variables increases the risk of false positive results the null hypothesis was rejected at P <0.01.

## Results

**Figure 1:**
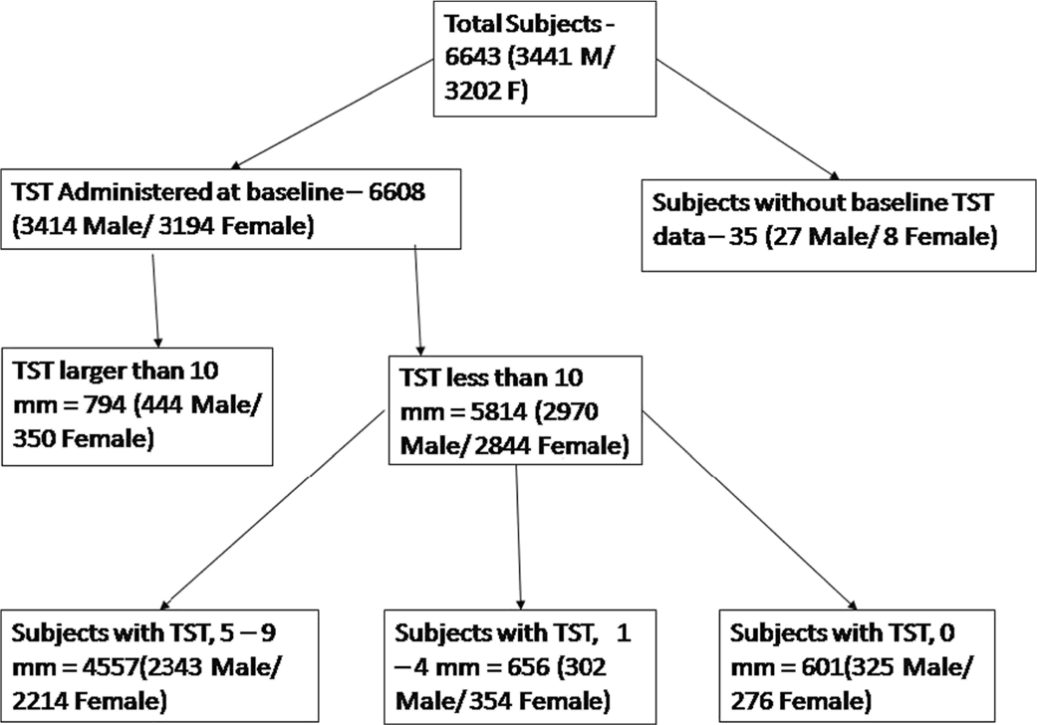
**Flow chart indicating participants who were enrolled into the study, and in whom the TST was administered and read.**

A total of 6643 adolescents were enrolled in the study, among them TST was administered to 6608 participants at baseline, 35 participants did not have a TST done; males and those who had illiterate mothers were more likely not to have had a TST done. Figure 1 shows a detailed flow chart of participants who were enrolled into the study and in whom the TST was administered and read for the present analysis. There were approximately equal proportions of girls and boys, and 80% (N = 5276) of the participants were 14 years or less in age. The proportion of socio-economically disadvantaged participants varied based on the parameter used as summarized in Table 1. Reported BCG vaccination was 87.1% and 62.7% of participants had a visible BCG scar (Table 1).

**Table 1:** **Sociodemographic characteristics of the study participants**

Characteristic ^a^	(N = 6608)
Sex	
Male	3414 (51.7)
Female	3194 (48.3)
Age	
11 – 12 years	1778 (26.9)
13 – 14 years	3498 (52.9)
15 – 16 years	1100 (16.6)
17 – 18 years	232 (3.5)
Caste	
Dalit/Harijan	1230 (18.6)
Others	5378 (81.4)
Religion	
Hindu	5851 (88.5)
Others	757 (11.5)
Education of mother*	
Illiterate	3190 (48.3)
Primary or Higher	3411 (51.7)
Education of father*	
Illiterate	1724 (26.1)
Primary or Higher	4856 (73.5)
Walls	
Brick	5238 (79.3)
Others^b^	1370 (20.7)
Cooking fuel	
LPG/Gas	819 (12.4)
Others^c^	5789 (87.6)
TB Contact*^d^	
Yes	44 (0.7)
No	6496 (99.3)
Unexplained cough ≥2 weeks	
Yes	40 (0.6)
No	6568 (99.4)
Unexplained weight loss ≥2 weeks	
Yes	27 (0.4)
No	6581 (99.6)
Unexplained fever ≥2 weeks	
Yes	18 (0.3)
No	6590 (99.7)
Other Symptoms^e^	
Yes	13 (0.2)
No	6595 (99.8)
Reported BCG immunization*	
Yes	5649 (87.1)
No	835 (12.9)
BCG Scar	
Yes	4142 (62.7)
No	2466 (37.3)
BMI- z Score*^f^	
Underweight (<− 2)	1991 (30.2)
Overweight (> +1)	181 (2.7)
Normal (−2 to +1)	4430 (67.1)
TST response	
0 mm	601 (9%)
1 – 4 mm	656 (10%)
5 - 9 mm	4557 (69%)
More than or equal to 10 mm	794 (12.0)

Reported as number and within parenthesis column percentages;

*Because data were not available on some individuals total numbers are lower than the number of subjects who participated in the study

^a^Cumulative response of both parent and subject has been considered in case of history of past TB, TB exposure, Symptoms suggestive of TB, Past disease history and past hospital admission.

^b^Includes walls made of Packed mud, Stone, Bamboo, Thatch, Wood.

^c^Includes Wood, Kerosene, Coal, Agricultural Residue as cooking fuel.

^d^Recent contact with a TB case at least for 8 hours a week; in the period of 3 months

^e^Other symptoms include unexplained Hemoptysis and night sweats for ≥2 weeks

^f^WHO cut offs 2007, Growth reference 5–19 years; calculated using WHO – ANTHRO software (version 3.2.2).

**Figure 2:**
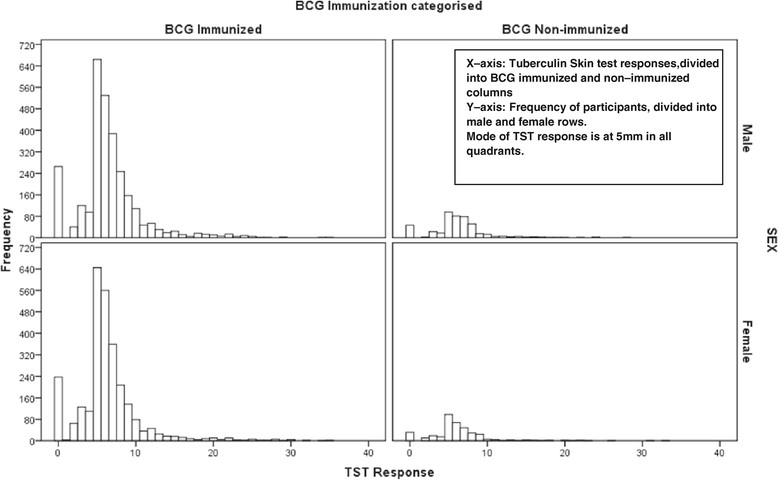
**Distribution of TST in males and females stratified for reported BCG immunization status.**

Among 6608 subjects, 601 (9.1%) had 0 mm, and 794 (12%) > = 10 mm responses respectively. Figure 2 shows that the distribution of TST responses is similar between those reported to be BCG immunized compared to those non – immunized; and between male and female participants.

The proportion of TST positive was higher in boys than in girls (AOR 1.19; 95% C.I. 1.02-1.38), in older participants (AOR 1.69; 95% C.I. 1.16 – 2.47 for 17 – 18 years and AOR 1.31; 95% C.I. 1.04 – 1.65 for 15 – 16 years), in those with a history of contact with TB (AOR 2.88; 95% C.I. 1.47 – 5.65) and in those with a reported history of BCG immunization (AOR 1.33; 95% C.I. 1.04 – 1.71). Increased odds were also seen in those who used LPG gas/electricity as a cooking fuel, often used as an indicator of relatively higher socio-economic status (AOR 1.26; 95% C.I. 1.02 – 1.57). Individuals who were underweight were less likely to be TST positive on a univariate analysis (OR 0.83; 95% C.I. 0.70 – 0.99), although this was not significant in the adjusted odds ratio (Table 2).

**Table 2:** **Sociodemographic, nutritional & clinical associations of the proportion of TST positive using a cutoff of ≥10 mm**

Characteristic ^a^	TST – 10 mm (N =7 94) (%)	TST <10 mm (N = 5814) (%)	P - value	OR (95% C.I)	Adjusted OR (95% C.I)
Sex					
Male	444 (13.0)	2970 (87.0)	0.01	1.21 (1.05 – 1.41)	1.19 (1.02 – 1.38)
Female	350 (11.0)	2844 (89.0)		1.00	
Age					
11 – 12 years	193 (10.9)	1585 (89.1)		1.00	
13 – 14 years	408 (11.7)	3090 (88.3)	0.38	1.08 (0.90 – 1.31)	1.09 (0.90 – 1.31)
15 – 16 years	153 (13.9)	947 (86.1)	0.01	1.33 (1.05 – 1.68)	1.31 (1.04 – 1.65)
17 – 18 years	40 (17.2)	192 (82.8)	0.004	1.71 (1.16 – 2.52)	1.69 (1.16 – 2.47)
Caste					
Dalit/Harijan	133 (10.8)	1097 (89.2)	0.15	0.87 (0.71 – 1.05)	
Others	661 (12.3)	4717 (87.7)		1.00	
Religion					
Hindu	714 (12.2)	5137 (87.8)	0.19	1.18 (0.91 – 1.51)	
Others	80 (10.6)	677 (89.4)		1.00	
Education of mother*					
Illiterate	372 (11.7)	2818 (88.3)	0.42	0.94 (0.81 – 1.09)	
Primary or Greater	420 (12.3)	2991 (87.7)		1.00	
Education of father*					
Illiterate	213 (12.4)	1511 (87.6)	0.56	1.05 (0.89 – 1.24)	
Primary or Greater	574 (11.8)	4282 (88.2)		1.00	
Walls					
Brick	633 (12.1)	4605 (87.9)	0.74	1.03 (0.86 – 1.24)	
Others^b^	161 (11.8)	1209 (88.2)		1.00	
Cooking fuel					
LPG/Gas	117 (14.3)	702 (85.7)	0.03	1.26 (1.01 – 1.56)	1.26 (1.02 – 1.57)
Others^c^	677 (11.7)	5112 (88.3)		1.00	
TB Contact*^d^					
Yes	13 (29.5)	31(70.5)	<0.001	3.10 (1.53 – 6.18)	2.88 (1.47 – 5.65)
No	774 (11.9)	5722 (88.1)		1.00	
Unexplained cough ≥2 weeks					
Yes	8 (20.0)	32 (80.0)	0.12	1.84 (0.84 – 4.00)	
No	786 (12.0)	5782 (88.0)		1.00	
Unexplained weight loss – 2 weeks					
Yes	2 (7.4)	25 (92.6)	0.46	0.59 (0.14 – 2.47)	
No	792 (12.0)	5789 (88.0)		1.00	
Unexplained fever – 2 weeks					
Yes	1 (5.6)	17 (94.4)	0.39	0.43 (0.06 – 3.24)	
No	793 (12.0)	5797 (88.0)		1.00	
Other symptoms^e^					
Yes	1 (8.3)	11 (91.7)	0.69	0.67 (0.03 – 4.94)	
No	793 (12.0)	5803 (88.0)		1.00	
BCG immunization*					
Yes	695 (12.3)	4954 (87.7)	0.01	1.36 (1.06 – 1.74)	1.33 (1.04 – 1.71)
No	78 (9.3)	757 (90.7)		1.00	
BCG Scar					
Yes	515 (12.4)	3627 (87.6)	0.18	1.11 (0.95 – 1.30)	
No	279 (11.3)	2187 (88.7)		1.00	
BMI - z Score*^f^					
Underweight (<− 2)	212 (10.6)	1779 (89.4)	0.03	0.83 (0.70 – 0.99)	
Overweight (> + 1)	26 (14.4)	155 (85.6)	0.46	1.17 (0.75 – 1.82)	
Normal (−2 to + 1)	555 (12.5)	3875 (87.5)		1.00	

Reported as number and within parenthesis row percentages; OR: Odds ratio; C.I: Confidence intervals; – - reference group; P value reported for univariate analysis; Adjusted odds ratios (Multivariate analysis) were calculated for those characteristics significant in the univariate analysis.

*Because data were not available on some individuals total numbers are lower than the number of subjects who participated in the study

^a^Cumulative response of both parent and subject has been considered in case of history of past TB, TB exposure, Symptoms suggestive of TB, Past disease history and past hospital admission.

^b^Includes walls made of Packed mud, Stone, Bamboo, Thatch, Wood.

^c^Includes Wood, Kerosene, Coal, Agricultural Residue as cooking fuel.

^d^Recent contact with a TB case at least for 8 hours a week; in the period of 3 months;

^e^Other symptoms include unexplained Hemoptysis and night sweats for ≥2 weeks

^f^WHO cut offs 2007, Growth reference 5–19 years; calculated using WHO – ANTHRO software (version 3.2.2).

Those who were more likely to be non responders to TST, included younger participants (AOR, 0.64; 95% C.I. 0.45 – 0.92 for 15 – 16 years age group and AOR 0.59; 95% C.I. 0.40 – 0.86 for 17 – 18 years group; the reference group is 11 – 12 years) and those with a recent unexplained history of weight loss (AOR 7.35; 95% C.I. 3.21 – 16.8). Those who were less likely to be non-responders included those living in houses with brick walls (relatively higher socio-economic class; AOR 0.74; 95% C.I. 0.61 – 0.91), living in houses where LPG/Gas was used as cooking fuel and those with a visible BCG scar (AOR 0.68; 95% C.I. 0.57 – 0.81). Underweight participants were more likely to be non-responders on a univariate (OR 1.23; 95% C.I. 1.03 – 1.48) but not multivariate analysis.

## Discussion

Our data delineate the pattern of TST responses in adolescent school-going children in South India. These data allow for the first comparison with data of other countries, since 2 TU PPD was used in this study, as opposed to 1 TU in the earlier studies in India [1],[2],[7],[14]. A small study in India had earlier suggested that TST reaction sizes were similar with both doses [6]. In the present study, 9.1% of participants had no response (0 mm) to the TST, while 12% had a TST response ≥10 mm.

Earlier studies conducted in Andhra Pradesh [2] showed a proportion of TST positive of about 9.7%, in younger children and using a higher cutoff of 20 mm at a 1TU dose. The lower proportion of TST positive (2% of participants i.e. N = 133) when we use the 20 mm cutoff in our data set could be because this study was limited to school-going children who may be healthier than those in the general population. It is plausible that we might have found higher TST positive rates, if all children both school going or school drop-outs were included. While it may appear contradictory that we found higher TST positive rates in older children, yet found lower rates of TST positivity compared to earlier studies which evaluated younger children, it is important to emphasise that the first finding reflects a within-study analysis while the second, is a cross-study comparison, where other variables may also have been operative.

The most significant determinants of TST responses are active or past TB disease [15] and recent exposure to TB disease [16]. The proportion of TST positive is associated with TB exposure, with increasing odds of the proportion of TST positive with increasing proximity of TB exposure [17],[18] with increasing age associated with an increased likelihood of TB exposure [19]-[21] and with male gender[22] These already documented associations were seen in our study as well. We also showed that lower socio-economic status was associated with a positive TST and underweight individuals were less likely to have a positive TST.

Data from Ghana, where a dose of 2 TU in 0.1 ml PPD RT23/Tween 80 was used, demonstrated an absence of a TST response in 41.9% of children with BCG scars and in 49.1% without BCG scars [23]. In the same study, TB infection rates using TST responses were shown to be similar in those with and without a BCG scar, irrespective of age of the individual [23]. In our study the proportion of TST positive was slightly higher in the children who had been BCG immunized compared to who had not received immunization. This is in contrast to studies that have indicated that TST results in vaccinated and non-vaccinated people are similar [24]. The reason for this discrepancy is not immediately clear. BCG vaccination may affect TST responses [25], although this is likely to be dependent on the interval between BCG vaccination and the TST [24],[26], and the age at TST [27]. Studies in India, where 1 TU PPD RT23 was used have indicated that BCG vaccination does not affect the TST responses in the 5 to 9 year age group [2],[28].

The non-responders (i.e. 0 mm) constituted 9.1% of participants. This proportion is lower than that found during the ARTI survey conducted by Chadha VK et al. during 2009 – 10 among 1 to 9 years children in the South zone of India (16.5% of participants had 0 mm reaction) [29]. We are unable to explain the differences between that reported by Chadha and our own data; however there are several plausible reasons – First, our study sample was older in age and, possibly therefore, more exposed to TB. Second, we used 2TU as opposed to 1TU used in the earlier surveys, this could have resulted in increased nonspecific (small) TST responses to, for instance, NTMs, although this is speculative. Third, our study population was restricted to those in schools, who could conceivably be more healthy than the entire population of children, including those out of school. While a comparison of 1TU and 2 TU tuberculin surveys are not ideal, given the different distribution patterns of the responses to these two doses (Chadha et al.) [6], we believe that reference to earlier work based on the 1 TU studies is important, since these are the only large population level data sets available in India.

Our non-response rate is also considerably lower than that reported in other settings such as Zambia (76%), South Africa (69%) [30], in local settings such as the crowded townships of Cape Town, South Africa (50.4%) [20], and even those in adjacent Bangladesh [31]. However, rates of HIV prevalence in our study setting were lower than many of the areas, particularly in Africa. The HIV prevalence among antenatal clinic attendees in Chittoor district (the study area) between 2010 – 11, was 0.5% to 1.0% and the prevalence of HIV in Andhra Pradesh state among adult was 0.90% [32]. Another plausible reason is that there may be a higher prevalence of NTM’s in parts of India leading to detectable but small TST responses.

TB infection determined by TST may be underestimated due to the poorer nutritional status in low socio-economic groups since malnutrition has been associated with lower TST responses [33]. However, another study in India did not find this association [14]. Differing indices of malnutrition across studies adds to the complexity of interpretation. In our sample, non-response to the TST was more often found among those with a lower age, recent unexplained weight loss and those without BCG scars. Those with objective low weight (using BMI for age cutoffs) also more often had a TST of 0 mm, but this was not significant in multivariate analysis (data not shown). While recent unexplained weight loss, would be subject to reporting bias and is less objective than anthropometric measurements, the two parameters also likely represent different aspects of nutritional status; the recent loss of weight reflecting a more acute phenomenon, while the BMI for age cutoff would also include more chronic nutritional deficits.

### Limitations of the study

The study was conducted in school-going children in a largely rural area of Southern Andhra Pradesh. School enrollment and drop-out rates vary across ages and gender [34]; being female, poverty and economic reasons were major factors associated with drop-outs [35]. Thus, our study sample is likely to have had a smaller proportion of the very poor compared to the general population. This may have limited our ability to demonstrate the effect of undernutrition on TST in the multivariate analyses that we performed. It is also plausible that the proportion of TST positive rate may be higher than we describe, given that school-going children may be generally healthier, although data to support this in this age group are lacking.

## Conclusions

Proportion of non-responders was similar than elsewhere in India but much lower than elsewhere in the world and some of them may be false negative, given the association with unexpected weight loss. Proportion of TST positivity was higher in those who had been exposed to TB and in children who had been BCG immunized, while a zero response was more likely in younger adolescents and those with recent weight loss. Besides the expected associations higher TST seemed to be associated with higher SES. Use of LPG/Gas may have an effect on TST itself, which needs further investigation.

## Authors’ contributions

Conceived and designed the experiments: MV, HMSG, DU. Performed the experiments: DU, NJ, SS, HMSG, MV. Analysed the data: DU, SS, MV. Contributed reagents/ material/ analysis tools: SS, SB, MV. Wrote the manuscript: DU, SV, HMSG, MV. Reviewed the manuscript and provided critical comments: NJ, SB, Involved in the development of the protocol of the larger study within which the present analysis is embedded as well as the implementation and quality control of the study: TBTSG. Worked as medical officer for the project and was part of the clinical data collection and supervision: DU. Principal Investigators for the project and were responsible for the conceptualization of the study question: HMSG, MV. All authors read and approved the final manuscript.

## Authors’ information

DU: Medical officer, St. John’s Emmaus TB Research Initiatives (SETRI), Palamaner, India.

SS: Biostatistician, St. John’s Research Institute, Bangalore, India.

NJ: Clinical Research Manager, St. John’s Emmaus TB Research Initiatives (SETRI), Palamaner, India.

SB: Gilead Sciences, Inc., Foster City CA, USA.

SV: KNCV Tuberculosis Foundation, The Hague, The Netherlands, and Academic Medical Centre Amsterdam, The Netherlands.

HMSG: Professor, Department of Clinical Science, Infection, Faculty of Medicine and Dentistry, University of Bergen and Department of Microbiology, Haukeland University Hospital, N-5021 Bergen, Norway.

MV: Professor, Physiology and Head of Health and Humanities , St. John’s Medical College and St. John’s Research Institute, Bangalore, India.

## The TB Trials Study Group (in alphabetic order)

1. Cardenas V, Aeras, Rockville, Maryland, USA.

2. Doherty M, Department of Infectious Disease Immunology, GlaxoSmithKline, Copenhagen, Denmark.

3. Grewal HMS, Department of Clinical Science, Infection, Faculty of Medicine and Dentistry, University of Bergen and Department of Microbiology, Haukeland University Hospital, N-5021 Bergen, Norway.

4. Hesseling AC, Department of Pediatrics and Child Health, Desmond Tutu TB Centre, Stellenbosch University, Cape Town, South Africa.

5. Jacob A, Emmaus Swiss Leprosy Project and Referral Hospital, Palamaner, Andhra Pradesh, India.

6. Jahnsen F, Department of Pathology, Oslo University Hospital, Rikshospitalet and University of Oslo, Nydalen, Oslo, Norway.

7. Kenneth J, Division of Infectious Diseases, St. John's Research Institute, Bangalore, Karnataka, India.

8. Kurpad AV, Division of Nutrition, St. John's Research Institute, Bangalore, Karnataka, India.

9. Lindtjorn B, Centre for International Health, Faculty of Medicine, University of Bergen, Bergen, Norway.

10. Macaden R, Infectious Diseases, St. John's Research Institute, Bangalore, Karnataka, India.

11. Nelson J, St. John’s Research Institute, Bangalore, Karnataka, India.

12. Vaz M, Health and Humanities, St. John’s Research Institute, Bangalore, Karnataka, India

## Authors’ original submitted files for images

Below are the links to the authors’ original submitted files for images. Authors’ original file for figure 1 Authors’ original file for figure 2

## Abbreviations

ARTI: Annual risk of tuberculosis infection
AOR: Adjusted odds ratio
BCG: Bacillus calmette guerin
BMI: Body mass index
C.I: Confidence intervals
HIV: Human Immunodeficiency Virus
M.tb: Mycobacterium *tuberculosis*
NTMs: Non-tuberculous mycobacteria
OR: Odds ratio
TST: Tuberculin skin test
TU: Tuberculin units
TB: Tuberculosis
WHO: World Health Organization

## References

[1] Chadha VK, Agarwal SP, Kumar P, Chauhan LS, Kollapan C, Jaganath PS, Vaidyanathan PS, Gopi PG, Unnikrishnan KP, Savanur SJ (2005). Annual risk of tuberculous infection in four defined zones of India: a comparative picture. Int J Tuberc Lung Dis.

[2] Chadha VK, Kumar P, Satyanarayana AV, Chauhan LS, Gupta J, Singh S, Magesh V, Lakshminarayana Ahmed J, Srivastava R, Suganthi P, Devi GU (2007). Annual risk of tuberculous infection in Andhra Pradesh, India. Indian J Tuberc.

[3] Kumar S, Chadhan VK, Jeetendra R, Kumar P, Chauhan LS, Srivastava R, Umadevi Kirankumar R (2009). Prevalence of tuberculous infection among school children in Kerala. Indian J Tuberc.

[4] Chopra K, Chadha VK, Ramachandra J, Aggarwal N (2012). Trend in annual risk of tuberculous infection in north India. PLoS One.

[5] Rao VG, Gopi PG, Yadav R, Subramani R, Bhat J, Anvikar AR, Sadacharam K, Tiwari BK, Gadge V, Bhondeley MK, Shukla GP, Ukey M, Jain S, Wares DF (2008). Annual risk of tuberculosis infection among tribal population of central India. Trop Med Int Health.

[6] Chadha VKJP, Nagaraj AV, Narayana Prasad D, Anantha N (2000). A comparative study of Tuberculin reactions to 1TU and 2TU of PPD – RT23. Indian J Tuberc.

[7] Chadha VK, Vaidyanathan PS, Jagannatha PS, Unnikrishnan KP, Mini PA (2003). Annual risk of tuberculous infection in the northern zone of India. Bull World Health Organ.

[8] Brennan MJ, Thole J (2012). Tuberculosis vaccines: a strategic blueprint for the next decade. Tuberculosis (Edinb).

[9] Brennan MJ, Fruth U, Milstien J, Tiernan R, de Andrade NS, Chocarro L (2007). Development of new tuberculosis vaccines: a global perspective on regulatory issues. PLoS Med.

[10] Arnadottir T, Rieder H, Trebucq A, Waaler H (1996). Guidelines for conducting tuberculin skin test surveys in high prevalence countries. Tuber Lung Dis.

[11] Kumar C, Patwari A (2013). Updated National Guidelines for Pediatric Tuberculosis in India, 2012: Some Unresolved Issues. Indian Pediatr.

[12] TB India 2008 [], [http://www.tbcindia.nic.in/pdfs/TB%20India%202009.pdf]

[13] Murthy M, Selvam S, Jesuraj N, Bennett S, Doherty M, Grewal HM, Vaz M (2013). Two-step tuberculin skin testing in school-going adolescents with initial 0–4 millimeter responses in a high tuberculosis prevalence setting in South India. PLoS One.

[14] Chadha VK, Jitendra R, Kumar P, Gupta J (2009). Umadevi: Relationship of nutritional status with tuberculin sensitivity. Indian J Pediatr.

[15] Richeldi L (2006). An update on the diagnosis of tuberculosis infection. Am J Respir Crit Care Med.

[16] Vijayasekaran D, Kumar RA, Gowrishankar NC, Nedunchelian K, Sethuraman S (2006). Mantoux and contact positivity in tuberculosis. Indian J Pediatr.

[17] Garcia-Sancho FM, Garcia-Garcia L, Jimenez-Corona ME, Palacios-Martinez M, Ferreyra-Reyes LD, Canizales-Quintero S, Cano-Arellano B, Ponce-de-Leon A, Sifuentes-Osornio J, Small P, DeRiemer K (2006). Is tuberculin skin testing useful to diagnose latent tuberculosis in BCG-vaccinated children?. Int J Epidemiol.

[18] Lienhardt C, Fielding K, Sillah J, Tunkara A, Donkor S, Manneh K, Warndorff D, McAdam KP, Bennett S (2003). Risk factors for tuberculosis infection in sub-Saharan Africa: a contact study in The Gambia. Am J Respir Crit Care Med.

[19] Radhakrishna S, Frieden TR, Subramani R (2003). Association of initial tuberculin sensitivity, age and sex with the incidence of tuberculosis in south India: a 15-year follow-up. Int J Tuberc Lung Dis.

[20] Wood R, Liang H, Wu H, Middelkoop K, Oni T, Rangaka MX, Wilkinson RJ, Bekker L-G, Lawn SD (2010). Changing prevalence of TB infection with increasing age in high TB burden townships in South Africa. Int J Tuberc Lung Dis.

[21] Middelkoop K, Bekker LG, Liang H, Aquino LD, Sebastian E, Myer L, Wood R (2011). Force of tuberculosis infection among adolescents in a high HIV and TB prevalence community: a cross-sectional observation study. BMC Infect Dis.

[22] Martinez AN, Rhee JT, Small PM, Behr MA (2000). Sex differences in the epidemiology of tuberculosis in San Francisco. Int J Tuberc Lung Dis.

[23] Addo KK, van den Hof S, Mensah GI, Hesse A, Bonsu C, Koram KA, Afutu FK, Bonsu FA (2010). A tuberculin skin test survey among Ghanaian school children. BMC Public Health.

[24] Menzies D (2000). What does tuberculin reactivity after bacille Calmette-Guerin vaccination tell us?. Clin Infect Dis.

[25] Joos TJ, Miller WC, Murdoch DM (2006). Tuberculin reactivity in bacille Calmette-Guerin vaccinated populations: a compilation of international data. Int J Tuberc Lung Dis.

[26] Neuenschwander BE, Zwahlen M, Kim SJ, Lee EG, Rieder HL (2002). Determination of the prevalence of infection with Mycobacterium tuberculosis among persons vaccinated against Bacillus Calmette-Guerin in South Korea. Am J Epidemiol.

[27] Wang PD (2008). Epidemiological trends of childhood tuberculosis in Taiwan, 1998–2005. Int J Tuberc Lung Dis.

[28] Chadha VK, Jaganath PS, Kumar P (2004). Tuberculin sensitivity among children vaccinated with BCG under universal immunization programme. Indian J Pediatr.

[29] Chadha VK, Sarin R, Narang P, John KR, Chopra KK, Jitendra R, Mendiratta DK, Vohra V, Shashidhara AN, Muniraj G, Gopi PG, Kumar P (2013). Trends in the annual risk of tuberculous infection in India. Int J Tuberc Lung Dis.

[30] Shanaube K, Sismanidis C, Ayles H, Beyers N, Schaap A, Lawrence KA, Barker A, Godfrey-Faussett P (2009). Annual risk of tuberculous infection using different methods in communities with a high prevalence of TB and HIV in Zambia and South Africa. PLoS One.

[31] Hossain S, Zaman K, Banu S, Quaiyum MA, Husain MA, Islam MA, Cooreman E, Borgdorff M, van Leth F (2013). Tuberculin survey in Bangladesh, 2007–2009: prevalence of tuberculous infection and implications for TB control. Int J Tuberc Lung Dis.

[32] National AIDS Control Programme Phase III; State Fact Sheet. 2012, AIDS Control GOI, New Delhi

[33] Pelly TF, Santillan CF, Gilman RH, Cabrera LZ, Garcia E, Vidal C, Zimic MJ, Moore DA, Evans CA (2005). Tuberculosis skin testing, anergy and protein malnutrition in Peru. Int J Tuberc Lung Dis.

[34] Kumar TV: Post Enumeration Survey of DISE 2011 (5% sample check). In Hyderabad: Centre for Equity and Social Development; NATIONAL INSTITUTE OF RURAL DEVELOPMENT; 2012.

[35] Reddy AN, Sinha S: School Dropouts or Pushouts? Overcoming Barriers for the Right to Education. CREATE Pathways to Access. Research Monograph No. 40. *Online Submission* 2010

