# Supplementary figures and images for: The tuberculin skin test in school going adolescents in South India: associations of socio-demographic and clinical characteristics with TST positivity and non-response
Source: BMC Infect Dis. 2014 Nov 18;14:571. doi: 10.1186/s12879-014-0571-7 (PMC4243729; doi:10.1186/s12879-014-0571-7)

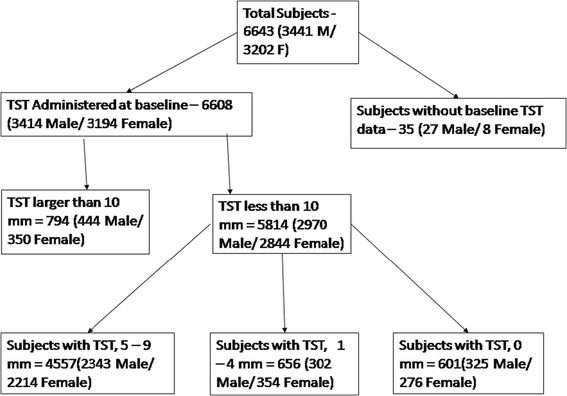

Supplement: Supplementary file 1 — Authors’ original file for figure 1 [file 12879_2014_571_MOESM1_ESM.gif]

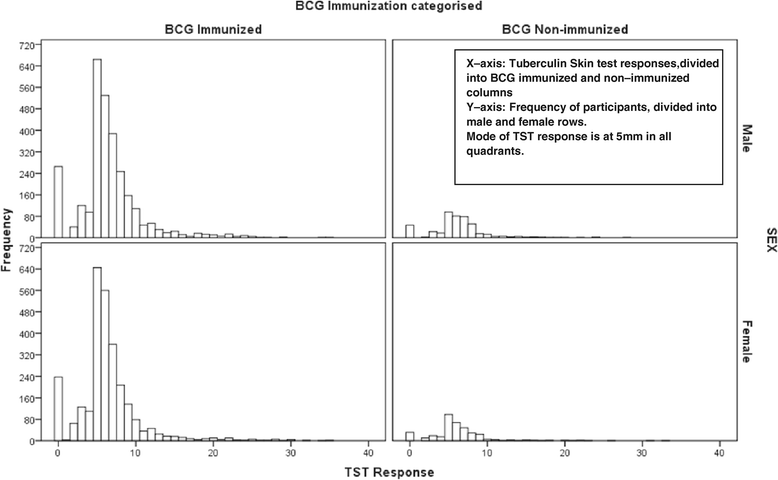

Supplement: Supplementary file 2 — Authors’ original file for figure 2 [file 12879_2014_571_MOESM2_ESM.gif]
